# GPs’ identification of patients with mental distress: a coupled questionnaire and cohort study from norwegian urban general practice

**DOI:** 10.1186/s12875-022-01865-x

**Published:** 2022-10-09

**Authors:** Mina P. Dahli, Ole R Haavet, Torleif Ruud, Mette Brekke

**Affiliations:** 1 Department of General Practice, Institute of Health and Society, Faculty of Medicine, University of Oslo, Kirkeveien 166 Fredrik Holst Hus, 0450 Oslo, Norway; 2 Division of Mental Health Services, Akershus University Hospital, Lørenskog, Norway; 3 Institute of Clinical Medicine, Faculty of Medicine, University of Oslo, Oslo, Norway; 4 General Practice Research Unit, Institute of Health and Society, Faculty of Medicine, University of Oslo, Oslo, Norway

**Keywords:** General practice, Psychological distress, Mental health, Diagnoses

## Abstract

**Background:**

Mental health problems are one of the leading causes of disease burden worldwide, and are mainly diagnosed and treated in general practice. It is unclear however, how general practitioners (GPs) identify mental health problems in their patients. The aim of this study was to explore how patients’ self-reported levels of mental distress correspond with psychological diagnoses made by their GPs, and associations with sex, age, number of consultations, and somatic symptom diagnoses.

**Methods:**

A questionnaire study coupled with retrospective and prospective cohort data from 553 patients aged 16–65 years in six GP offices in Oslo, Norway during 21 months in 2014–2016.

**Results:**

We found that 73.3% of patients with self-reported high levels of mental distress versus only 13.3% of the patients with low levels of mental distress had received a psychological diagnosis (p < 0.01). We found an increase in number of consultations for the group with high levels of mental distress regardless of having received a psychological diagnosis (p < 0.01). There was also an increase in number of somatic symptoms (p = 0.04) and higher number of females (0.04) in this group. 35% of patients had received one or more psychological diagnosis by their GP. Mean CORE-10 score, being female and a high number of consultations was associated with having received a psychological diagnosis. In the adjusted analyses high CORE-10 score and a high number of consultations still predicted a psychological diagnosis.

**Conclusions:**

We found a clear association between self-reported mental distress and having received a psychological diagnosis amongst the participants, and the probability for being identified increased with increasing levels of mental distress, and increasing number of visits to their doctor. This suggests that GPs can identify patients with high levels of mental distress in general practice in an adequate way, even though this can sometimes be a complex issue.

**Trial registration:**

Trial registration The main study was retrospectively registered in ClinicalTrials.gov on August 10 2019 with identification number NCT03624829.

**Supplementary Information:**

The online version contains supplementary material available at 10.1186/s12875-022-01865-x.

## Background

Mental health problems are one of the leading causes of disease burden worldwide [1]. These problems range from mild distress to severe psychiatric illness. How these patients are identified and what characterizes them is thus important to explore, so measures can be taken to provide adequate and high-quality care. General practice is usually the first point of contact with the health care system, and where the largest portion of these patients are treated [2, 3]. General practitioners (GPs) are therefore in a good position to provide health care to this group of patients.

Many studies over several decades have pointed towards GPs’ lack of correctly identifying and treating mental health problems in their patients [4–9]. Most of these studies look at the extent to which GPs can diagnose defined mental illnesses, such as depression or anxiety disorders [10, 11]. Fewer studies have looked at how GPs identify general mental distress in their patients and we have not found any studies from within the last decade.

Mental distress refers to significant emotional upset that is common to a range of psychological and psychiatric conditions. A greater proportion of primary care patients suffer distress than suffer depression [6]. Recognition of mental distress is strongly associated with management and outcome [12]. Recognized as compared to non-recognized cases are more likely to receive adequate health care, and have better outcomes in terms of both mental health and social functioning [12]. The cost of failing to diagnose and treat mental health problems can be dramatic, as these issues result in increased utilization of health care and lower work participation [13–15].

Improving GPs’ knowledge of their patients’ histories and circumstances, as well as the patients’ ability and space to communicate the entirety of their problems are crucial components of improving health care quality [4, 5, 16, 17]. Facilitating these elements within the time limits of a primary care setting and in a cost-effective manner would have an enormous impact on several levels of the health care system [18]. Some have proposed that psychosocial assessments should be integrated into routine primary care, and studies have shown that patients are positive towards this type of assessment intervention [18].

In this study, we wanted to explore whether self-reported mental distress amongst patients is related to the probability of having received a psychological diagnosis in consultation with a GP. We wanted to compare self-reported mental distress through a questionnaire survey, coupled with diagnostic data from electronic medical records from patients GPs. Finally, we wanted to explore whether patients’ age, sex, number of consultations and number of somatic symptom diagnoses influenced on this probability of receiving a psychological diagnosis.

## Methods

### Study population and participants

This study is part of a larger research project: Shared Care and Usual Health Care for Mental and Comorbid Health Problems, a cluster-randomized controlled intervention study on the impact on patients and health care by a shared care between GPs and mental health care services [19–23]. This study is based on two datasets from the baseline data in the main project. Firstly, a questionnaire study was performed during two weeks in 2015 at six GP office centers in Groruddalen, Oslo, Norway. All patients aged 16–65 years entering the office centers during these two weeks were invited to fill in a questionnaire (CORE-10) before their appointment with a doctor. All participants were given written information about the one-page questionnaire, available in Norwegian and English. They gave written consent before participation. Secondly, we collected electronic medical records from all patients aged 16–65 from these office centers 12 months retrospectively and nine months prospectively from when the questionnaire study was performed. The prospective period could not be extended to 12 months as it would interfere with the intervention in the main study [19].

The upper age limit of 65 years was set as it is the lower age limit for geriatric patients, and they are followed up in separate departments in specialized mental health care and therefore not included in the main study.

In Norway, there is a system where all patients have the right to a GP according to a list system administered by the state through local municipalities. Over 99% of all Norwegian citizens are registered with a GP.

## Questionnaire data

The questionnaire consisted of CORE-10, a comprised version of ten items on psychiatric symptoms drawn from the CORE-OM questionnaire, developed to measure mental distress for use in primary health care services [24, 25]. CORE-10 is evaluated as a reliable and valid instrument that is practical to use with people presenting with common mental health problems in primary care settings [25, 26]. We collected 845 questionnaires in total. Of these, 215 questionnaires were excluded as they were incomplete, or the patients were outside of the age range (16–65 at inclusion). Another 77 were excluded because of incomplete data or participants not being part of the cohort of registered patients at the offices. This left 553 questionnaires included in this study. This is presented in Fig. 1 in the Supplementary Material 1.

## Cohort data

Electronic patient records from all patients aged 16–65 years at inclusion were collected, 12 months retrospectively and later nine months prospectively from the time that the questionnaire study was performed. All registered contacts were included, as were age, sex, date of contact, type of contact, International Classification of Primary Care 2nd edition (ICPC-2) diagnoses [27], and tariffs (reimbursement codes) used by the GPs. A computer program was developed by the firm Mediata AS for this project to extract data from the different GP office centers. This included data from 16 845 patients with direct consultations with GPs. Phone contacts, prescriptions, meetings, and other types of contacts not specific to contact with a GP directly were not included. We coupled these cohort data to the questionnaire data for those patients participating in the questionnaire study and had given written consent. Later we extracted the same variables nine months prospectively, but now only for the patients included in the questionnaire study.

## Data management

The questionnaire data and consent forms are kept in a safe server at Akershus University Hospital and the cohort data in a Service for Sensitive Data platform administrated by the University of Oslo.

## Data analyses

For the “number of somatic symptoms” variable, we collected all symptom diagnoses (00–29) from the ICPC-2 chapters; General and unspecified, Digestive, Musculoskeletal, Neurological, and Social problems, and aggregated them, so each diagnosis given was registered only once. Descriptive statistics in the form of frequencies and percentages were used to describe categorical variables, whereas numerical data were described by means and standard deviations.

Mean differences between groups were accessed using the independent t-test, whereas associations between categorical variables were analyzed using a Chi-square test. We performed binary logistic regression looking at factors that were associated with having a psychological diagnosis. The variables used in the binary regression were; Age, sex, mean CORE-10 score, mean number of consultations and number of somatic symptom diagnoses. Each variable was analyzed adjusting for all the other variables in this group. All analyses were performed using STATA SE 16 (StataCorp, College Station, TX) and IBM SPSS Statistics 25 (Armonk, NY), and the significance level was set at p = 0.05.

## Results

### Patient characteristics

A total of 553 patients were included in the study. Of them, 384 (69.4%) were women and 169 (30.6%) were men. The data collection process is presented in Fig. 1 in the Supplementary Material 1. Mean age for the whole group was 43.1 [95% CI: 41.9, 44.2] years, 42.0 [95% CI: 40.7, 43.4] years for women and 45.5 [95% CI: 43.4, 47.5] years for men. The patients included had 11.6 ± 7.6 mean number of consultations with their GP during the period, 12.0 ± 7.7 for the women, and 10.6 ± 7.4 for the men.

The number of somatic symptom diagnoses ranged from zero (160 patients) to 11 (one patient), the mean number of somatic symptom diagnoses was 1.7 ± 1.7, for the women 1.8 ± 1.8, and 1.4 ± 1.5 for the men.

## Level of mental distress

The mean CORE-10 score for all patients was 14.8 ± 5.6; the mean score for the women was 15.2 [95% CI: 14.6, 15.8], and the mean score for the men was 14.0 [95% CI: 13.1, 14.8]. Table 1 shows the distribution of CORE-10 scores in the sample.

**Table 1:** Distribution of CORE-10 scores for 553 patients aged 16–65 in Norwegian general practice in 2015–2016

CORE-10 Score	Total n (%)	Men (%)	Women (%)
Healthy (0–5)	18 (3.3)	8 (4.7)	10 (2.6)
Low level problems (> 5–10)	102 (18.4)	36 (21.3)	66 (17.2)
Mild psychological distress (11–15)	202 (36.5)	61 (36.1)	141 (36.7)
Moderate distress (16–20)	141 (25.5)	43 (25.4)	98 (25.5)
Moderately severe (21–25)	67 (12.1)	17 (10.1)	50 (13.0)
Severe psychological distress (> 26–40)	23 (4.2)	4 (2.4)	19 (5.0)
SUM	553 (100.0)	169 (32.9)	384 (67.1)

## Mental distress and GP assigned psychological diagnosis

**Fig. 1:**
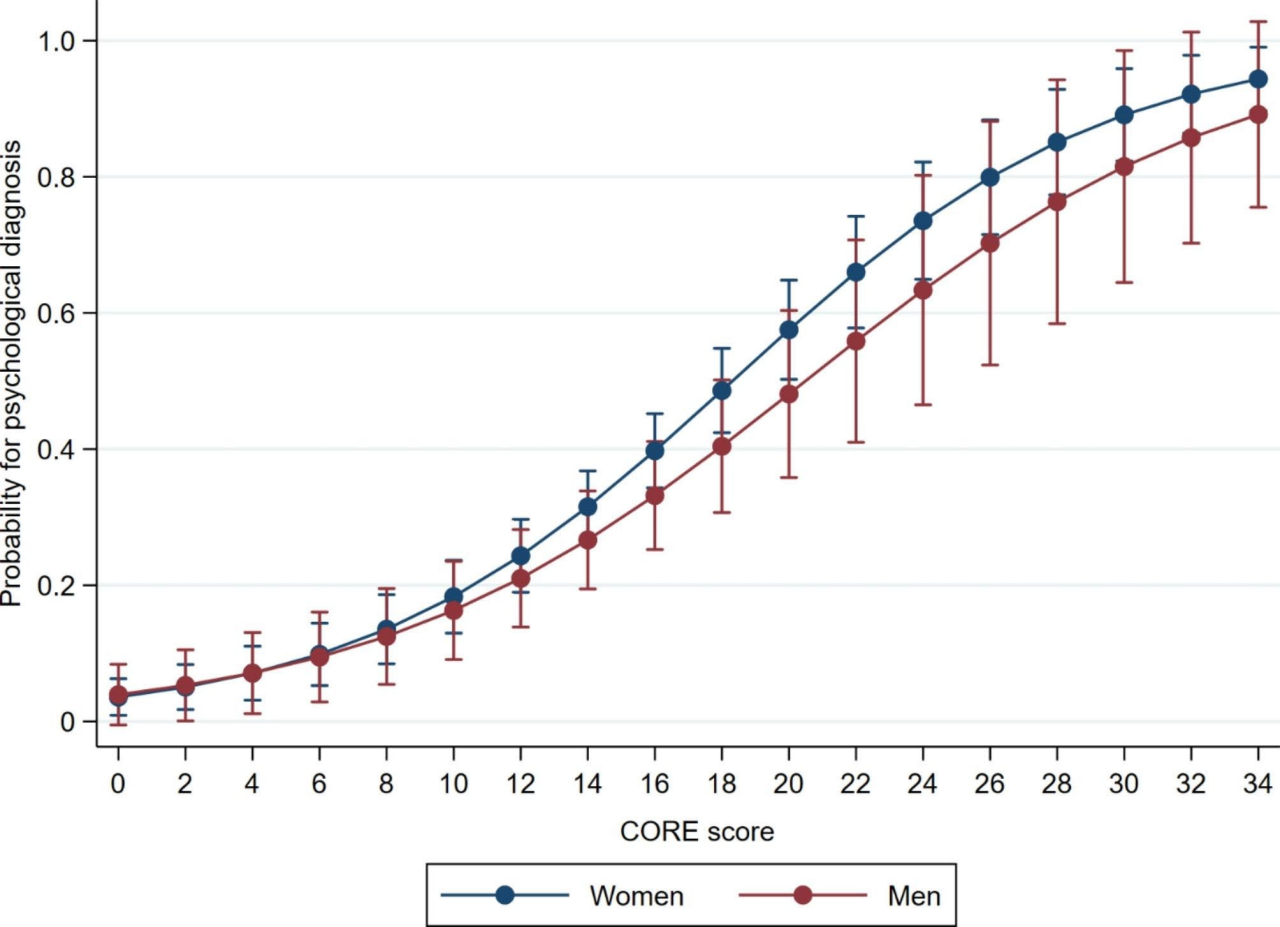
Associations between psychological diagnosis and CORE-10 scores among 553 patients aged 16–65 years in Norwegian general practice in 2015–2016 (95% CI)

We found a clear association between the level of self-reported mental distress and having received a psychological diagnosis amongst the participants. Figure 1 shows the marginal effects obtained from a binary logistical regression model describing the probability of having a psychological diagnosis with increasing CORE-10 scores for the participants in this study.

Of the patients with the highest levels of distress (CORE-10 score 21–40) (n = 90), 74.3% had received a psychological diagnosis versus only 13.3% of the patients with low levels of distress (CORE-10 score 0–10) (n = 120) (p < 0.001). Comparing these two groups we found a significant difference in the number of consultations (p < 0.01), the number of somatic symptom diagnoses (p = 0.04) with both being higher in the high levels of distress group as shown in Table 2. We found no significant differences in age, but being female was a significant factor (0.04) between the two groups.

**Table 2:** Characteristics of 210 patients aged 16–65 years with severe versus low self-reported mental distress in Norwegian general practice

Covariates	Severe mental distress (CORE-10 scores 21–40)	Low mental distress (CORE-10 scores 0–10)	*P*-value
Sex: n (%)			0.04
Women	69 (32.9)	76 (36.2)	
Men	21 (10.0)	44 (21.0)	
Age (years): mean (SD)	44.7 (12.5)	42.3 (13.3)	0.81
Number of consultations: mean (SD)	15.9 (9.4)	9.10 (6.1)	< 0.01
Psychological diagnosis (%)	66 (73.3)	24 (13.3)	< 0.01
Number of somatic symptom diagnoses: mean (SD)	1.8 (1.7)	1.3 (1.5)	0.04

## Patient characteristics and probability of receiving a psychological diagnosis

35% of patients received one or more psychological diagnosis in the period, either as a symptom (152 patients), disorder (95 patients), or both (51 patients). Comparing patients with or without a psychological diagnosis in the material, we found that sex, mean number of consultations, and mean CORE-10 score was associated with having received a psychological diagnosis, as shown in Table 3.

**Table 3:** Characteristics of 553 patients aged 16–65 years with or without a psychological diagnosis in Norwegian general practice in 2015–2016

Covariates	Psychological Diagnosis (n = 196)	No Psychological Diagnosis (n 357)	*P*-value
Sex: n (%)			0.04
Women	147 (26.6)	237 (42.9)	
Men	49 (8.9)	120 (21.7)	
Age (years): mean (SD)	43.4 (12.8)	42.9 (14.1)	0.63
Number of consultations: mean (SD)	14.3 (8.7)	10.1 (6.5)	< 0.01
CORE-10 score: mean (SD)	17.9 (5.7)	13.1 (4.8)	< 0.01
Number of somatic symptom diagnoses: mean (SD)	1.8 (1.9)	1.6 (1.6)	0.10

The results of a binary logistic regression analysis looking at factors associated with having a psychological diagnosis are presented in Table 4. We found that mean CORE-10 score was still a highly significant factor (p < 0.01) when adjusting for age, sex, number of consultations and number of somatic symptom diagnoses received. The number of consultations was still a highly significant factor (p < 0.01) when adjusting for age, sex, mean CORE-10 score and number of somatic symptom diagnoses. When adjusting for mean CORE-10 score, number of consultations and age, sex (being female) was no longer a significant factor giving increased probability for a psychological diagnosis compared to males. These results presented as odds rations, show that each additional consultation and each unit increase in CORE-10 score significantly increased the likelihood of having a psychological diagnosis by 7% (OR 1.07) and 17% (OR 1.17) respectively.

**Table 4:** Probability for a psychological diagnosis in 553 patients aged 16–65 years in Norwegian general practice in 2015–2016

Covariates	Unadjusted		Adjusted	
	OR (95% CI)	*P*-value	OR (95% CI)	*P*-value
Sex (ref: Men)				
Women	1.52 (1.03, 2.25)	0.04	1.31 (0.84, 2.04)	0.23
Age in years	1.00 (0.99, 1.02)	0.64	0.99 (0.98, 1.01	0.37
Mean number of consultations	1.08 (1.05, 1.10)	< 0.01	1.07 (1.04, 1.11)	< 0.01
Number of somatic symptom diagnoses	1.09 (0.99,1.21)	0.08	0.95 (0.83, 1.08)	0.40
CORE-10 Clinical score	1.19, (1.15,1.24)	< 0.01	1.17 (1.12, 1.22)	< 0.01

The CORE-10 questionnaire has one item regarding suicidal thoughts (question 6: Do you have thought about ending your life?). Nine patients rated themselves as having these thoughts “most of the time”, five of these had received a psychological diagnosis.

## Discussion

We found a clear dose-response relationship between the level of self-reported mental distress and the probability of having received a psychological diagnosis by a GP amongst the participants. This is coherent with other literature [4, 28]. We also found that patients with higher levels of mental distress have more consultations than patients with low levels of mental distress, and the probability for having received a psychological diagnosis increased with the number of contacts, when adjusted for the level of mental distress, age, sex and number of somatic symptom diagnoses.

The strength of this study is that we were able to connect self-reported mental distress in patients with their full electronic medical records from 12 months before and nine months after the survey, including all contacts and all diagnoses. This gives a comprehensive picture of these patients’ contact with their GP.

There are several limitations to this study. Mainly, we could only couple the cohort data to patients participating in the questionnaire study. We could not collect data on the other patients declining participation, therefore limiting our information on the comparability between the patients who wanted to participate and those who did not. All patients entering the doctors’ offices during the 2 week inclusion period was invited to participate. We could have simply counted the patients declining, but we did not. There is a possible selection bias as the patients were recruited by the researchers in the waiting room before a doctor’s appointment. We can theorize that patients with high levels of mental distress would be more likely to say “no” to participation, and patients with low levels of mental distress more likely to participate. This would skew our results in the direction of lower overall mental distress amongst the participants. This study was performed in three boroughs in Groruddalen in Oslo. These are suburbs with a high level of immigrants and low socioeconomic level compared to other areas of Oslo and Norway as a whole. We can also assume that patients with little Norwegian or English language skills would be more likely to decline participation, and therefore reduce the number of immigrant patients even though all information was available in English and Norwegian.

The CORE-10 questionnaires ask participants to include how they have felt the last week. We do not know their stress debut, duration, whether it was a short reactive state, related to a specific life event, or as a part of a larger psychiatric condition or other physical illness. We do not know whether patients were in early stages, or at the tail end of their illness and follow up. This is why we included a large cohort to explore patterns both before and after the survey was performed.

There are several factors to consider when exploring the complexities of psychological diagnosis in general practice. GPs are tasked with determining the nature of patients’ mental distress and providing appropriate care. Sophisticated consulting skills are required to differentiate a wide range of symptoms from a complex narrative in a short amount of time, as the patient’s experience and description of symptoms do not follow the body-mind divide that characterizes the classification of disease in the health care system today [29].

Distinctions between “normal” mental distress and psychiatric disorders are not always clear-cut; they depend on how these disorders are conceptualized, for both patients and doctors [16, 30]. Patients’ specific beliefs about their presenting symptoms play an important role in predicting GPs’ recognition and treatment of mental distress [4, 5]. Not all patients with less severe symptoms of psychological distress may want to or need to be treated [31]. Some patients may present symptoms due to short-term adjustment disorders that might not require intervention, but overlap with symptoms of mental disorders. Patients may disguise or underplay their symptoms because of the stigma of disclosure or differing beliefs about their presenting symptoms may affect how they are disclosed [4, 5, 32]. This is particularly apparent during initial visits to the GP before a trusting relationship has been established [32].

Patients tend to present with somatic presentations of mental distress in general practice [33]. Most GPs are cautious not to miss a life-threatening organic condition or serious somatic illness when presented with varying somatic symptoms [33]. They will tend to investigate somatic symptoms first, and risk becoming focused on possible organic disease instead of considering mental illness when patients present with somatic distress [29, 32].

What is the “gold standard” for diagnosing different mental health disorders in primary care? Different instruments for “screening” and diagnosing mental illness show differing results when compared [34]. There are also differing perspectives amongst GPs and psychiatric specialists in this area [35–40]. A Danish qualitative study from 2014 found that for depression, psychiatrists regarded the diagnosis as a pragmatic and agreed construct, and did not question its validity. GPs considered depression as more of a “gray area” and questioned the clinical utility in general practice. GPs were also more skeptical about instruments that they felt could be misleading [37]. For patients that present with symptoms that are perhaps not included as part of a psychiatric disorder, GPs may refrain from setting a psychiatric diagnosis, even when they suspect one [39, 41].

Increasing recognition of mental health problems does not necessarily correspond to better outcomes for patients, as increased recognition only helps if GPs and other parts of the health care system have the skills and resources to deliver adequate interventions once the patients are identified [42].

## Conclusion

Our study shows that GPs are largely able to identify mental distress in patients, but there are many unknown factors when it comes to identifying mental distress and mental health problems in general practice still. We need further research as this group of patients and their care has such a large impact on our health care system.

## Electronic supplementary material

Below is the link to the electronic supplementary material.

Supplementary Material 1

## Data Availability

The datasets used and analyzed during this study are available from the project’s principal investigator (TR) on reasonable request.

## List of abbreviations

CORE-10: Clinical Outcomes in Routine Evaluation 10.
GP: General practitioner.
ICPC-2: International Classification of Primary Care, 2nd edition.
