# Supplementary material for: GPs’ identification of patients with mental distress: a coupled questionnaire and cohort study from norwegian urban general practice
Source: BMC Prim Care. 2022 Oct 9;23:260. doi: 10.1186/s12875-022-01865-x (PMC9549632; doi:10.1186/s12875-022-01865-x)

**APPENDIX**

**Figure 1**. Collection of questionnaire data for 553 patients in Norwegian general practice in 2015-2016


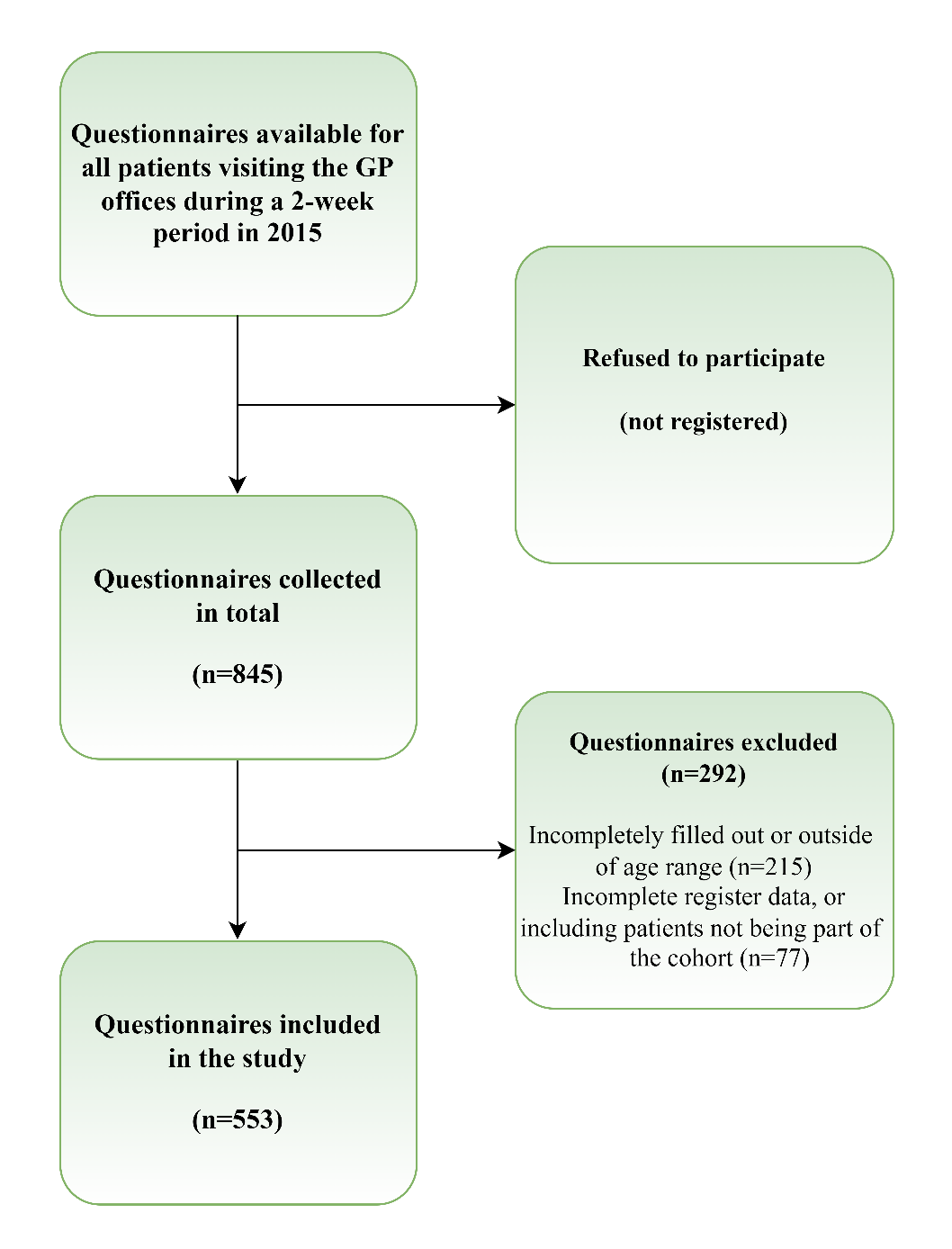

Supplement: Supplementary file 1 — Supplementary Material 1 [file 12875_2022_1865_MOESM1_ESM.docx]
